# The Association of Relationship Status and Sex-Life Satisfaction With Body Dissatisfaction and Drive for Muscularity in Male Weight-Lifters

**DOI:** 10.3389/fpsyg.2018.02759

**Published:** 2019-01-17

**Authors:** Catharina Schneider, Julia Bartuschka, Martin Voracek, Kristina Hennig-Fast

**Affiliations:** ^1^Department of Applied Psychology: Health, Development, Enhancement, and Intervention, Faculty of Psychology, University of Vienna, Vienna, Austria; ^2^Department of Basic Psychological Research and Research Methods, Faculty of Psychology, University of Vienna, Vienna, Austria; ^3^Department of Psychiatry and Psychotherapy, Evangelisches Krankenhaus Bielefeld, Bielefeld, Germany

**Keywords:** drive for muscularity, body dissatisfaction, romantic relationship, sexual satisfaction, weight lifting

## Abstract

Relationship status and sexuality are linked to body image concerns, but research on the connection to men’s drive for muscularity (DfM) is scarce. Extreme DfM can lead to a pathological preoccupation with muscularity and problematic eating/exercising behavior. This study investigated the relation of relationship status, relationship duration, and satisfaction with sex-life in weight-lifting men via an online survey (*N* = 270). Using cross-sectional data, we found that single weight-lifting men and those dissatisfied with their sex-life were more dissatisfied with their muscularity and showed stronger DfM than those in a relationship and satisfied men. Longer relationship duration was associated with less dissatisfaction with muscularity and less DfM while relationship satisfaction was not. Thus, being in a relationship and sexual satisfaction are related to less body dissatisfaction and DfM. Further research should use dyadic study designs to investigate both partners exercising and eating behavior in relation to each other.

## Introduction

Body dissatisfaction and drive for muscularity (DfM) in men represent a relatively recent field of study. DfM is defined as “the extent to which individuals desire to achieve the muscular ideal and act in ways to maximize the likelihood that they will become muscular” (McCreary, 2012, p. 561). With regard to male body image it has been proven useful to divide body dissatisfaction into two sub-categories: dissatisfaction with body fat and dissatisfaction with muscularity (Tylka, 2011).

Especially bodybuilding and weight-lifting men seem to be prone to be dissatisfied with their muscularity and show higher degrees of DfM than men not involved in these types of sports (Blouin and Goldfield, 1995). It seems self-evident that men participating in a sport that aims for muscles and strength display higher degrees in DfM. At the same time, these higher degrees can go along with psychological problems, such as exercise dependence, the misuse of anabolic steroids, eating pathology, and low self-esteem (Chittester and Hausenblas, 2009).

A variety of studies found negative relations of DfM with body image-related factors such as body shame and body dissatisfaction (Mustapic et al., 2015) or socio-cultural factors such as internalization of male body ideals (Daniel and Bridges, 2010) and social body comparisons (Stratton et al., 2015). Furthermore, negative associations with psychological factors or symptoms were found, such as anabolic steroid misuse (Litt and Dodge, 2008), lower self-esteem (Murray et al., 2013), depression (Edwards et al., 2014), and ultimately muscle dysmorphia (MD, Robert et al., 2009). For a more in-depth review of DfM and associated risk factors, see, e.g., Edwards et al. (2014).

Muscle dysmorphia is a recently described phenomenon, representing a pathological preoccupation with ones’ own muscularity (Pope et al., 2000b). It is positioned within the chapter on body dysmorphic disorders in the Diagnostic and Statistical Manual-5 (DSM-5; American Psychological Association, 2013) and is characterized by a strong desire to increase lean muscle mass as well as the belief of being insufficiently muscular (Murray and Touyz, 2013). Main features are a strict exercise regimen and high-protein diets (Pope et al., 2000b).

Although DfM is only one aspect representing MD, it might be the defining one. It was suggested, that DfM can be understood as a continuum, unproblematic in low/moderate intensity, but potentially harmful with possible pathological outcomes like MD in its extreme form (Robert et al., 2009). Although not all individuals with high degrees of DfM develop MD, some of the attitudinal and behavioral outcomes associated with MD are consistent with DfM (Robert et al., 2009) and DfM has been identified as a precursor for the development of MD (Olivardia et al., 2000). Thus, with higher rates of DfM and higher risk for MD, bodybuilding and weight-lifting men are of special interest to investigate male body image and associated factors.

While body image is associated with many aspects of life, romantic/intimate relationships and sexuality have been found to display complex links with body satisfaction and DfM. Existing research investigated these links with various outcomes (Tom et al., 2005; Filiault, 2007; McCabe and McGreevy, 2010; Goins et al., 2012; Swami et al., 2014).

While a general positive connection of relationship status and duration with body satisfaction has been reported (Tom et al., 2005), more specific findings associated partners’ satisfaction with and feedback on ones’ body with men’s body satisfaction (Goins et al., 2012; Goldsmith and Byers, 2016). Men were more satisfied with their bodies when they perceived their partners to be satisfied (Goins et al., 2012). Usually partners’ feedback was complimentary and focused on health rather than physical attractiveness (McCabe and McGreevy, 2010). Furthermore, positive feedback on the body was also related to confidence, self-acceptance, and sexual fulfillment (Goldsmith and Byers, 2016).

Tom et al. (2005) concluded that, although body dissatisfaction exists in married and single persons alike, this dissatisfaction is not as important for married individuals. Thus, long-lasting and satisfying relationships may help to moderate the impact of unrealistic body ideals. Being single, on the contrary, was found to be related to more proneness to internalization of appearance ideals and DfM (Giles and Close, 2008). According to sexuality, studies found that higher degrees of sexual intimacy between partners were connected to greater body satisfaction (Goins et al., 2012) and thus might function as a protective factor against body dissatisfaction and maybe also DfM.

Prior research findings are based on college or community samples. Since bodybuilding and weight-lifting men seem to be at higher risk for negative psychological outcomes of body dissatisfaction and DfM (Blouin and Goldfield, 1995), we chose, in line with suggestions by McCreary (2012) to investigate this special population. The three aims of our study were thus, (1) to compare single vs. in-relationship weight-lifting men and (2) weight-lifting men satisfied vs. dissatisfied with the frequency of their sexual contacts for their dissatisfaction with their muscularity and body fat, as well as their DfM. We hypothesized that men in relationships and those satisfied with the frequency of their sexual contacts will display less dissatisfaction with muscularity and body fat as well as lower DfM. Furthermore, we investigated (3) the association of relationship duration and satisfaction with dissatisfaction with muscularity and body fat as well as DfM, assuming that longer relationships and higher satisfaction with relationship goes along with less dissatisfaction with muscularity and body fat as well as lower DfM.

## Materials and Methods

### Participants and Procedure

Participants were recruited via announcements in different online panels and social media groups for the German-speaking weight-lifting and bodybuilding community. They were provided with a link to the survey. After giving informed consent, the questionnaire took approximately 30 min. Participants were treated in accordance with the Declaration of Helsinki and attended voluntarily and anonymously. After completion of the questionnaire, they were invited to take part in a lottery for two 50€ vouchers for dietary supplements.

Five hundred and thirty-eight individuals provided consent to participate in this study. Of these, 230 persons had to be excluded for not finishing the questionnaire. Also, two participants who were under 18 years of age, 15 men who did not exercise regularly and 21 women were excluded. The final sample consisted of 270 regularly weight-lifting males. Sample composition details are shown in Table 1.

**Table 1:** Descriptive statistics for demographic and anthropometric variables.

Variable	Mean	*SD*	Min	Max	Variable		%
Age	26.25	7.51	18	51	Nationality	German	67
Weight (kg)	84.96	13.08	60	135		Austrian	30.4
Height (m)	1.81	0.07	1.60	2.03		Other	2.6
BMI	25.96	3.43	18.22	41.21	Education level	Below high-school	26.3
Body fat (%)	14.26	4.74	4	40		High-school	38.9
FFMI	22.14	2.66	16.52	36.2		Higher education	34.8
Relationship duration (years)	4.9	5.5	0.08	30.4	Relationship status	In relationship	56.7
Duration training (years)	5.2	5.7	0.08	34		Single	43.3
Training per week	3.9	1.09	2	7	Sexual orientation	heterosexual	96.3
Duration training per session (min)^∗^	80.41	23.7	30	180		homosexual	0.4
						bisexual	3
					Sexual satisfaction	satisfied	31.9
						dissatisfied (wanting more)	64.8
						dissatisfied (wanting less)	3.3


*BMI = body mass index, FFMI = fat-free mass index, ^∗^three extreme outliers of 0, 3, and 5 min of training had to be excluded.*

The (self-reported) average body mass index (BMI) was 25.96, classifying the sample as (slightly) overweight (a BMI from 19.00 to 24.99 refers to the normal weight range while a BMI from 25.00 to 29.99 refers to overweight). Body fat percentage averaged 14.3% which is below the Austrian average of 22.3% (Elmadfa et al., 2012). The fat-free mass index (FFMI, characterizing a persons’ degree of muscularity, Pope et al., 2000a) was 22.14, classifying the participants as more muscular than the average male American/European college student (Pope et al., 2000b).

### Measures

#### Sociodemographic Measures and Exercise-Related Variables

After consent was gained, sociodemographic data (e.g., nationality, age, relationship status), anthropometric data (e.g., reported height, weight, and body fat percentage) as well as workout-related behavior (e.g., years of exercise, average training time) was collected.

#### Relationship Satisfaction

Relationship satisfaction was assessed with the 10-item partnership questionnaire (Kliem et al., 2012) on 4-point scales (0 = *never/rarely*; 4 = *very often*). Cronbach’s α was 0.81. According to Kliem et al. (2012) we classified quality of a relationship as poor with a score ≤ 12.

#### Satisfaction With Frequency of Sexual Contact

Participants responded to two questions regarding their satisfaction with frequency of sexual contact, phrased analogously to items on the sexual desire inventory (Spector et al., 1996). Participants indicated how often they would have liked to engage in sexual activity in the last month and how often they actually had engaged in it using an 8-point scale (1 = *not at all*; 8 = *more than once a day*). Dissatisfaction with the frequency with sexual encounters was defined by deviation from 0.

#### Dissatisfaction With Body Fat and Muscularity

The Bodybuilder Image Grid, Scaled (BIG-S, Hildebrandt et al., 2004) is a bi-dimensional silhouette figure rating scale depicting 30 male figures showing different degrees of body fat and muscularity. Muscle and body fat dissatisfaction are represented by the contrast between ratings of current and ideal body figures. Higher scores (positive and negative deviations from 0) on either dimension represent the desire to become either leaner or more muscular. Test–retest reliability was previously reported to be 0.84–0.93.

#### Drive for Muscularity

Drive for muscularity was assessed with the German version of the Drive for Muscularity Scale (DMS, Waldorf et al., 2014). The 15-item questionnaire (6-point scale; 1 = *always*, 6 = *never*) is summed up to a total value. Additionally, two subscales, *muscularity-related attitudes* and *muscularity-related behavior* are calculated. Sample reliability for the scales are shown in Table 2.

**Table 2:** Mean comparisons of body-related variables.

Variable	Single		In relationship				Sex. dissatisfaction		Sex satisfaction			
	
	*M*	*SD*	*M*	*SD*	*p*	$\eta_{p}^{2}$	*M*	*SD*	*M*	*SD*	*p*	$\eta_{p}^{2}$
Dissatisfaction with body fat	-0.84	0.10	-1.02	0.07	0.144	0.01	-0.92	0.07	-0.94	0.09	0.867	0.00
Dissatisfaction with muscularity	0.86	0.06	0.67	0.05	0.014	0.02	0.86	0.05	0.67	0.07	0.023	0.02
Muscularity-related attitudes	3.73	0.13	3.35	0.10	0.023	0.02	3.79	0.10	3.29	0.14	0.003	0.03
Muscularity-related behavior	3.25	0.09	3.37	0.07	0.297	0.00	3.44	0.07	3.18	0.10	0.032	0.03


## Results

We used multivariate analysis of variance (MANOVA) with subsequent discriminant analysis for the hypotheses regarding group differences on body dissatisfaction and DfM. Furthermore, we used correlational analysis for the hypotheses regarding associations of relationship duration and satisfaction with body dissatisfaction and DfM.

A MANOVA (variables: single vs. relationship and variables: satisfied vs. dissatisfied with frequency of sexual contacts) showed significant differences between single vs. in relationship, Λ = 0.93, *F*(4, 263) = 4.86, *p* = 0.001, $\eta_{p}^{2}$ = 0.07, and sexually satisfied vs. dissatisfied weight-lifting men Λ = 0.96, *F*(4, 263) = 2.71, *p* = 0.031, $\eta_{p}^{2}$ = 0.04 and no significant interaction between the two variables.

The MANOVA was followed up with discriminant analysis for each of the two groups, revealing one discriminant function each. Predictor variables were dissatisfaction with muscularity, dissatisfaction with body fat, and the two DfM subscales muscularity-related attitudes, and muscularity-related behavior. For the groups single vs. in relationship, the discriminant function revealed a significant association between groups and predictors, Λ = 0.93, χ^2^(4) = 18.48, *p* = 0.001, canonical *R^2^* = 0.07, accounting for 7% of between group variability. Analysis of the structure matrix showed two significant predictors, namely muscularity-related attitudes (0.644) and dissatisfaction with muscularity (0.511). The cross validated classification showed that overall 62.2% were correctly classified. As can be seen in Table 2, single men were more dissatisfied with their muscularity and showed higher muscularity-related attitudes than men in relationships.

For the groups of sexually satisfied vs. dissatisfied men the discriminant function also significantly differentiated the two groups, Λ = 0.94, χ^2^(4) = 17.35, *p* = 0.002, canonical *R^2^* = 0.06. Again, only muscularity-related attitudes (0.866) and dissatisfaction with muscularity (0.776) could be classified as useful predictors. Here, 61.9% of all cases were correctly classified.

Significant negative correlations were found between duration of relationship and dissatisfaction with muscularity, *r*(151) = -0.17, *p* = 0.036, muscularity-related attitudes, *r*(151) = -0.24, *p* = 0.003, muscularity-related behavior *r*(151) = -0.23, *p* = 0.005 but not with dissatisfaction with body fat. Controlling for age these correlations became insignificant. We found no significant correlations between relationship satisfaction and body dissatisfaction or the DfM subscales.

## Discussion

The main aim of this study was to examine the connection of (a) relationship status, (b) duration, (c) satisfaction, and (d) sexual satisfaction with body dissatisfaction and DfM in weight-lifting men. To measure DfM we used the two subscales of the DMS, namely *muscularity-related attitudes* and *muscularity-related behaviors*.

Generally our findings support the notion that single men are more dissatisfied and more prone to attain an ideal body (Giles and Close, 2008) than men in relationships. This might be due to the assumption that meeting a male body ideal and thus enhancing ones’ physical attractiveness, may help improve ones’ chances of finding a potential sexual/romantic partner.

More specifically, we found that muscularity-related attitudes and dissatisfaction with muscularity differentiated single weight-lifting men from those in a relationship. Singles were more dissatisfied with their muscularity and showed more muscularity-related attitudes than those in relationships, while muscularity-related behavior and dissatisfaction with body fat could not differentiate between the groups. This could be due to differences between attitudes and behavior regarding muscularity in weight-lifting men. While single weight-lifting men might spent more time thinking about their goal to become more muscular than those men in relationships, they do not necessarily train or diet more to attain this goal.

This finding is only partly in line with those of Giles and Close (2008) from college or community samples and might hint at a difference between weight-lifting men and those not regularly engaging in weight-lifting. Giles and Close (2008) found that muscularity-related attitudes and behavior were predicted by media exposure mediated though internalization of male body ideals. Therefore, the desire to become more muscular goes along with related behavior to attain a certain body ideal in men not regularly engaging in weight-lifting. For weight-lifting men this does not necessarily apply since they already exercise and diet to a higher degree than the average person.

In conclusion, our findings lend support to the hypothesis that single men, regardless of physical activity, are more prone to a male body ideal than men in a relationship, but that weight-lifting single men contrary to men not regularly engaging in weight-lifting do not necessarily act accordingly because they already are more muscular than average. According to Pope et al. (2000a) with a FFMI of 22 our sample classified as *noticeably* muscular, and thus more muscular than college samples which usually display a FFMI of 19–20.

Regarding dissatisfaction with muscularity our findings are in agreement with Tom et al. (2005) who found that long-lasting and satisfying relationships can mitigate the impact of ideal body images. For weight-lifting men this seems to be applying in a similar manner regarding dissatisfaction with muscularity. Again, being in a relationship might decrease the importance of physical attractiveness (muscularity), because one no longer has to attract a potential partner. In contrast to Tom et al. (2005) we could not find a connection with duration of the relationship. When controlling for age the correlations became insignificant. Since age was found to be associated with dissatisfaction with muscularity and DfM before (Schneider et al., 2016) further research seems to be required. Here, it seems to be of special interest to highlight the connection of age and duration of relationship, since the older a person is the higher the probability for a long-term relationship gets.

Contrary to our assumptions based on other findings (Tom et al., 2005), we found no significant correlations between relationship satisfaction and body dissatisfaction or muscularity-related attitudes and behaviors. This could be due to relatively homogenous statements of relationship satisfaction. Using the cut-point suggested by Kliem et al. (2012), 90.2% of participants stated to be satisfied with their relationship.

Regarding our second aim we found that muscularity-related attitudes and dissatisfaction with muscularity could discriminate between the groups of men being satisfied with the frequency of sexual encounters, irrespective of relationship status, and those not satisfied. The former were more content with their muscularity and showed lesser degrees of muscularity-related attitudes than the latter. This is in line with Goins et al. (2012), who found that higher degrees of sexual intimacy goes along with higher body satisfaction. Although sexuality is usually assumed to be part of intimate relationships, it is relatively independent from relationship status, and therefore, might be equally important for men’s body image concerns as relationships. Future research should investigate both aspects in relation to each other.

Despite the novelty of this study, limitations included the use of cross-sectional data and questioning only men instead of both partners. Also, a more in-depth approach, investigating the satisfaction with ones’ sex life (regarding e.g., intimacy and body related aspects) could be informative. Furthermore, since our sample reported to be predominantly heterosexual, a comparison with a sample of bi- and homosexual men could be interesting. Lastly, the results of this study cannot be generalized to men who are not weight-lifting, as this group displays special physical characteristics.

There has not been much research on the relation of relationship status, sexuality and body satisfaction in weight-lifting men. Therefore, our results can give first insight in connections of relationship and sexuality with body image concerns for men.

## Ethics Statement

This study was carried out in accordance with the recommendations of the Ethics Committee of the University of Vienna. The study was conducted as an online survey recruiting participants in the Internet via social media. Therefore, an ethic committees’ approval was not obtained. Before the start of the survey, participants were informed about the purpose of the study and they were able to drop out of it at any point with their data being deleted. Participation was voluntarily and anonymously. All participants gave written informed consent in accordance with the Declaration of Helsinki.

## Author Contributions

CS and JB designed and conducted the study in consultation with KH-F, and analyzed the data with assistance and contributions from MV and KH-F. CS drafted the manuscript with contributions from all other authors, who all read and approved to final manuscript.

## Conflict of Interest Statement

The authors declare that the research was conducted in the absence of any commercial or financial relationships that could be construed as a potential conflict of interest.
